# Efficacy and safety of herbal medicine combined with acupuncture in pediatric epilepsy treatment: A meta-analysis of randomized controlled trials

**DOI:** 10.1371/journal.pone.0303201

**Published:** 2024-05-09

**Authors:** Hong-Wen Su, Hsiao-Tien Chen, Chia-Li Kao, Kuo-Chuan Hung, Yao-Tsung Lin, Ping-Hsin Liu, Chien-Ming Lin, I-Wen Chen

**Affiliations:** 1 Department of Chinese Medicine, Chi Mei Medical Center, Tainan City, Taiwan; 2 Department of Anesthesiology, E-Da Hospital, I-Shou University, Kaohsiung City, Taiwan; 3 Department of Anesthesiology, Chi Mei Medical Center, Tainan City, Taiwan; 4 Department of Anesthesiology, E-Da Dachang Hospital, I-Shou University, Kaohsiung City, Taiwan; 5 Department of Anesthesiology, Chi Mei Medical Center, Liouying, Tainan City, Taiwan; Universiti Sains Malaysia, MALAYSIA

## Abstract

**Objective:**

To evaluate the efficacy and safety of herbal medicine and acupuncture combination for pediatric epilepsy treatment.

**Methods:**

Databases were searched from their interception until October 2023 to identify randomized controlled trials focusing on the therapeutic efficacy of herbal medicine-acupuncture combination (intervention group) for pediatric epilepsy. The primary outcome was the risk of treatment failure, whereas the secondary outcomes included the risk of post-treatment electroencephalogram (EEG) abnormalities and adverse events. Subgroup analyses were conducted based on the type of herbal compound formulas. Meta-regression analysis was conducted to examine the influence of patient demographics and clinical history on the therapeutic efficacy of herbal medicine-acupuncture combination for pediatric epilepsy. To assess the cumulative evidence, trial sequential analysis (TSA) was performed.

**Results:**

The analysis included 10 trials involving a total of 882 pediatric patients. Meta-analysis revealed that the intervention group had a lower risk of treatment failure than the control group (risk ratio [RR] = 0.3, 95% confidence interval [CI]: 0.19–0.47, *P*<0.00001, *I*^*2*^ = 0%, 10 trials). Subgroup analyses showed that therapeutic efficacy was consistent among the different herbal compound formulas. Meta-regression analysis revealed that the efficacy of the treatments did not significantly vary with patient age, male sex, and duration of seizure history. TSA suggested that herbal medicine-acupuncture combination exerted a robust and conclusive effect on seizure treatment. Although the combined used of herbal medicine and acupuncture was not associated with a lower risk of post-treatment EEG abnormalities (RR = 0.82, 95%CI:0.6–1.11, *P* = 0.2, 3 trials), the risk of adverse events was reduced (RR = 0.27, 95%CI:0.18–0.41, *P*<0.00001, 4 trials).

**Conclusion:**

The meta-analysis suggested that combined use of herbal medicine and acupuncture is a promising and safe clinical approach for pediatric epilepsy treatment. Further large-scale studies are necessary to conclusively determine the efficacy and safety of herbal medicine and acupuncture in pediatric epilepsy treatment.

## 1. Introduction

Epilepsy, a prevalent neurological disorder characterized by recurrent seizures, affects around 50 million people globally [1]. Of this population, nearly 10.5 million (25%) are children below 15 years old [2]. The onset of epilepsy in childhood is particularly concerning owing to its potential to disrupt developmental trajectories across neurological, cognitive, behavioral, motor, and weight domains [3]. Compared with that in adults, epilepsy in children has profound implications for brain development, educational attainment, injury risk, psychosocial challenges, and long-term life prospects [4–6]. Thus, effective management of epilepsy during childhood is a critical health priority. Antiepileptic drugs (AEDs) serve as the cornerstone of pediatric epilepsy management, with ketogenic diets and surgical interventions providing additional treatment avenues [7]. However, AEDs are associated with adverse effects such as fatigue, mood fluctuations, aggression, sleep issues, and cognitive impairments, including memory and attention deficits [8]. These side effects are particularly pronounced when polytherapy is used rather than single-drug treatments [9]. Notably, common AEDs, such as valproate, phenobarbital, carbamazepine, phenytoin, and oxcarbazepine, have been associated with cognitive side effects in children to varying extents [10]. This has increased the interest of healthcare providers in complementary therapies that can be integrated with conventional Western medical practices.

Traditional Chinese Medicine (TCM), including herbal medicine and acupuncture, has played a pivotal role in health management [11, 12]. Some studies have shown therapeutic synergies when acupuncture and herbal medicine are combined across various disease models compared with either therapy alone. For example, evidence indicates that this integrative approach improves chronic conditions, such as chronic prostatitis [13], and modulates metabolic disorders, such as gouty arthritis and atopic dermatitis [14, 15]. In pediatrics, the combination of herbal medicine and acupuncture has also shown substantial benefits for conditions such as tic syndrome and cerebral palsy, improving both physical and cognitive function [16, 17].

Herbal medicine remedies have been effective in treating adult epilepsy, particularly in decreasing seizure frequency and normalizing electroencephalogram (EEG) patterns [12, 18, 19]. Acupuncture, on the other hand, has also traditionally been used in adult epilepsy care [20], and recent studies suggest that it could be beneficial for adult patients with epilepsy when combined with Western medicine [21]. Although various studies have demonstrated the benefits of herbal medicine or acupuncture in treating pediatric epilepsy [22–31], the therapeutic efficacy and potential side effects of their combination have not yet been systematically evaluated. The absence of a systematic evaluation emphasizes the need for meta-analyses dedicated to examining the combined use of herbal medicine and acupuncture for treating pediatric epilepsy. Conducting such meticulous reviews would significantly improve our understanding of the efficacy and safety of herbal medicines and acupuncture in clinical practice.

## 2. Method

The protocol for this study was registered and is available for consultation in the International Prospective Register of Systematic Reviews. In addition, the conduct and reporting of this study strictly complied with the Preferred Reporting Items for Systematic Reviews and Meta-Analyses guidelines (registration number: CRD42023474478).

### 2.1. Search strategies and data sources

An exhaustive literature search was conducted to identify randomized controlled trials (RCTs) that investigated the efficacy and safety of the combined use of herbal medicine and acupuncture for pediatric epilepsy treatment. The search encompassed major databases, including PubMed, MEDLINE, Embase, and the Cochrane Central Register of Controlled Trials, spanning from their inception dates until October 20, 2023. The Clinical Trials gov database was also searched to ensure the inclusion of ongoing or unpublished studies. The search strategy combined keywords with medical subject headings (such as MeSH terms in Medline), incorporating the following keywords: (“Traditional Chinese medicine” or “Chinese herbal medicine” or “Herbal medicine” or “herbal” or “herb” or “botanical”) and (“Acupuncture” or “acupuncture therapy” or “needle acupuncture” or “auricular acupuncture”) and (“epilepsy” or “epilepsies” or “epilepsy’s”) and (“pediatrics” or “paediatrics” or “infant” or “paediatric” or “pediatric”). Furthermore, to ensure that no pertinent studies were overlooked, the search was extended to Google Scholar, Chongqing VIP Database, China National Knowledge Infrastructure (CNKI), Chinese Biomedical Literature Database, and Wanfang Data. The CNKI (http://www.cnki.net/) was used for Chinese studies, applying the terms “Dian-Xian” (epilepsy), “Xiao-Er” (pediatric), and combinations of “Zhongyao,” “Zhong-yi,” or “Zhen-jiu” (all signifying traditional Chinese medicine) with “Suiji” (randomized). To identify additional studies, the reference lists of published systematic reviews and included RCTs were screened. No restrictions were implemented on language, sample size, publication date, or country of origin. Details on the search strategies used for the database queries can be found in S1 Table.

### 2.3. Inclusion criteria and study selection

The following PICOS criteria were used to screen eligible trials: (P) participants (children aged <18 years with epilepsy), (I) interventions (combined use of herbal medicine and acupuncture for epilepsy treatment), (C) comparisons (standard care or use of conventional AEDs), (O) outcomes (risk of treatment failure, post-treatment EEG abnormalities, and adverse events), and (S) study design (only RCTs were included).

The following criteria were used to exclude ineligible studies: (a) inclusion of both adults and children, (b) use of herbal medicine or acupuncture in the control group, and (c) unavailability of full-text or epilepsy-related outcomes.

After removing duplicate records, the titles and abstracts of eligible studies were screened by two independent reviewers. Then, full-text articles were obtained and reviewed by the same reviewer to determine the inclusion criteria. In cases of disagreement, a third reviewer was consulted to facilitate discussion and reach consensus.

### 2.4. Data extraction

Data extraction was performed independently by two reviewers using a standardized data extraction form. The extracted data included the first author’s name, publication year, baseline characteristics of participants (e.g., age), number of study populations, herbal medicine formula, details of the acupuncture, and types of control. The clinical characteristics and outcomes (e.g., treatment failure rate) of each study were collected. In cases of divergence, a third reviewer made a final decision to resolve any discrepancies.

### 2.5. Outcomes and definition

The primary outcome of this study was the risk of treatment failure. This was defined by a decrease of less than 50% in seizure frequency from baseline, no improvement or deterioration in symptoms, therapy discontinuation due to severe side effects, or continuous high frequency of seizures without clinical or EEG amelioration. As for the secondary outcomes, we evaluated the risk of EEG anomalies post-treatment and the occurrence of adverse events. EEG abnormalities were specifically defined as a reduction of less than 50% in the length or number of epileptiform discharges on post-treatment EEGs relative to the baseline measures.

### 2.6. Quality of included studies

The methodological quality of the selected trials was independently evaluated by two reviewers using the Cochrane risk of bias (RoB2) tool. The RoB2 tool includes several critical domains for the identification of possible biases, including the randomization process, deviations from intended interventions, missing outcome data, outcome measurements, and selection of reported results. Discrepancies between the reviewers were addressed through discussion, and when an impasse occurred, a third reviewer was consulted to reach a consensus.

### 2.7. Certainty of evidence

The Grading of Recommendations Assessment, Development, and Evaluation (GRADE) approach was employed to evaluate the overall quality of evidence in this study. The GRADE guidelines encompass the following five domains: risk of bias, inconsistency, indirectness, imprecision, and potential publication bias. These domains collectively contribute to the assessment of evidence quality and are classified into four levels: high, moderate, low, and very low.

### 2.8. Data analysis

Review Manager (RevMan) version 5.4.1 was used to conduct the meta-analysis. Variables were categorized as risk ratios (RRs) with 95% confidence intervals (CIs). A *I*^*2*^ value exceeding 50% indicates significant heterogeneity. Given the variation in clinical settings, a random effects model was used. To determine the possible sources of heterogeneity, subgroup analyses were conducted based on the herbal compound formula (i.e., Ping Gan vs. Xi Feng vs. Din Xian). A sensitivity analysis was conducted by sequentially excluding each trial one at a time to evaluate the robustness of the results. When at least 10 trials or datasets were included, a funnel plot was used to detect potential publication bias. Furthermore, meta-regression analysis was conducted using Comprehensive Meta-Analysis (Version 4, Biostat, Englewood, NJ, USA) to investigate the influence of patient demographics and clinical history on the efficacy of the combined use of herbal medicine and acupuncture in the treatment of pediatric epilepsy. The following covariates were considered: patient age, male sex, and duration of seizure history. *P* < 0.05 was considered to indicate statistical significance.

To account for the risk of type I errors due to repetitive testing of accumulating data in systematic reviews and meta-analyses, trial sequential analysis (TSA) was employed. TSA provides a method for determining whether the available evidence in a meta-analysis is sufficient and conclusive. It adjusts the thresholds for statistical significance based on the amount of accumulated data, providing a more stringent criterion for statistical significance than the traditional methods. For the analysis, the required information size was established based on an anticipated relative risk reduction of 20%, type I error of 5%, and power of 80%.

## 3. Results

### 3.1. Study selection

**Fig 1:**
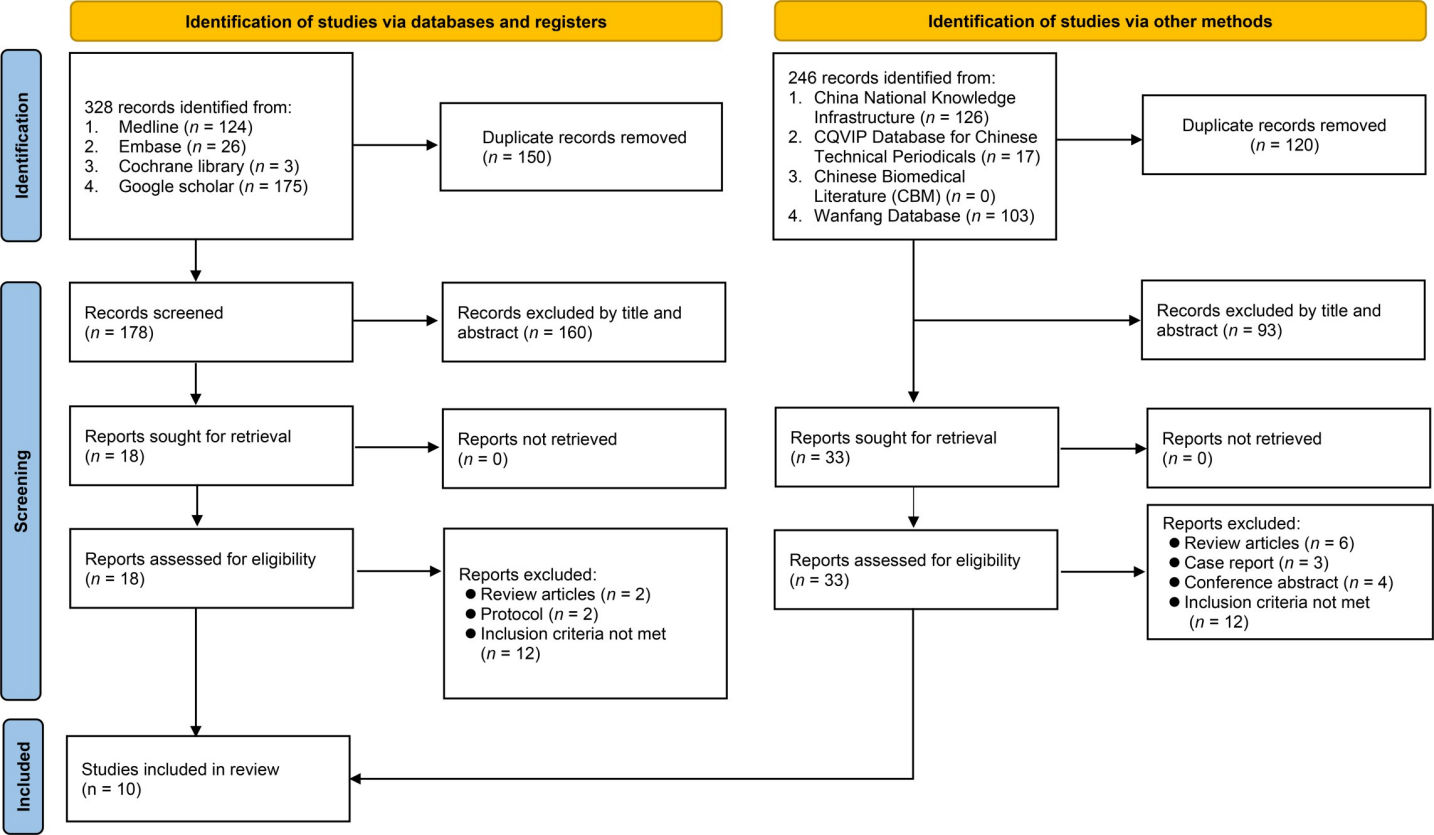
Screen process for studies.

From the initial 328 records sourced from the 4 electronic databases, 150 were removed due to duplication. Of the remaining 178 records, 160 were deemed ineligible after the title and abstract screening. Detailed full-text screening of the 18 remaining studies led to further exclusion of 16 reports, leaving 2 RCTs that satisfied the inclusion criteria for this study (Fig 1). Further searches in databases such as CNKI led to the identification of eight additional RCTs. Consequently, a total of 10 RCTs, all conducted in China and published between 2001 and 2019, were included in the analysis [22–31].

### 3.2. Characteristics of the studies

Table 1 presents the characteristics of the 10 included studies. These studies enrolled children aged 1–17 years, with most focusing on preschool to elementary school ages (1–11 years). In six studies, the patients’ mean age ranged from 2.1 to 7.8 years [22–25, 29, 31], whereas in four studies, the mean age was not specified [26–28, 30]. Male patients outnumbered female patients in all studies, accounting for 51.9% to 71.1% of the total number of samples. The average duration of seizure history before the treatment initiation widely ranged from 4.0 to 10 years across studies that reported this data [23–25, 28–31]. The total sample size was relatively small, comprising 882 children. In the studies analyzed, the intervention groups consisted of patients mainly treated with three herbal compound formulas, namely, Xi Feng, Ping Gan, and Ding Xian Tang, combined with various single-herb prescriptions. The details regard the herbal medicine and acupuncture are available in Tables 2 and 3. In the review of antiseizure protocols in the included RCTs, a total of 22 single-herb prescriptions were identified. Among these, Gastrodia elata Bl. (Tian-Ma), Scorpio (Quan-Xie), and Cicadae Periostracum (Jiang-Can) emerged as the most commonly used herbs (Table 2). In all studies, the duration of acupuncture was 30 min (Table 3). The control groups received conventional antiepileptic medications, including valproic acid, topiramate, phenytoin, phenobarbital, carbamazepine, oxcarbazepine, and clonazepam. All studies were conducted in China.

**Table 1:** Characteristics of studies (n = 10).

Author (year)	Age range (year)	Mean age (year)	Male gender (%)	History of seizure (years)	*n* ^a^	Study duration (Day)	Groups		Outcomes	Country
							Intervention	Control		
Hsu (2004)	1∼12	4.8	58.1	na	64 vs. 60	70	Ding Xian Tang Acupuncture	valproic acid, Phenytoin, Carbamazepine	1	China
Lee (2014)	1∼8	2.1 vs. 2.3^a^	51.9	4.3 vs 4.4^a^	53 vs. 53	56	Ping Gan formula Acupuncture	Topiramate	1,3	China
Liu (2014)	1∼8	2.3	53.6	4.2	56 vs. 56	60	Ping Gan formula Acupuncture	Topiramate	1,3	China
Lu (2016)	5∼14	2.49	52.7	8.26	55 vs. 55	56	Ping Gan formula Acupuncture	Topiramate	1,3	China
Lu (2018)	1∼12	Na	61.7	na	30 vs. 30	70	Ding Xian Tang Acupuncture	valproic acid	1	China
Ma (2001)	3∼16	<5y: 43% vs. 76.7% ^a^	68.3	na	30 vs. 30	18	Xi Feng Capsules Acupuncture	Carbamazepine, valproic acid, Phenobarbital, Clonazepam, Phenytoin	1,2	China
Su (2019)	3∼14	7.7 vs. 7.8^a^	54.7	7.8 vs. 8.1^a^	32 vs. 32	35	Xi Feng Capsules Acupuncture	valproic acid	1	China
Xiong (2003)	1∼17	<6y: 81%	71.1	4-10y: 57.8%	30 vs. 30	22	Xi Feng Capsules Acupuncture	Carbamazepine	1,2	China
Yang (2013)	3∼11	2.7 vs. 2.4^a^	52.6	7.6 vs. 7.4^a^	58 vs. 58	NA	Ping Gan formula Acupuncture	valproic acid, Phenobarbital, Clonazepam, Phenytoin	1,3	China
Zhong (2015)	6∼14	na	64.3	9.1 vs. 9.7^a^	35 vs. 35	NA	Ding Xian Tang Acupuncture	valproic acid, Oxcarbazepine, Carbamazepine	1,2	China

^a^present as intervention vs control groups; NA: not available

Outcome

1. Treatment failure rate

2. Post-treatment electroencephalography (EEG) abnormalities

3. Adverse effect

**Table 2:** Details of Chinese herbal medicine (n = 10).

Author (year)	Formula	Herbal medicine	Dose	The number of daily doses/ routes of administration
Hsu (2004)	Ding Xian Tang	*Gastrodia elata*, *Bulbus fritillariae*, *Arisaematis rhizoma preparatum*, *Rhizoma Pinelliae*, *Citri Reticulatae Pericarpium*, *Poria cocos*, *Poria pararadicis*, *Radix Salviae liguliobae*, *Acori graminei Rhizoma*, *Polygala tenuifolia*, *Ophiopogon japonicus*, *Buthus martensii Karsch*, *Bombyx batryticatus*, *Succinum*, *Cinnabaris*, *Bambusa tuldoides Munro*, *Zingiber officinale*, *Radix Glycyrrhizae*	NA	take once every other day/ oral
Lee (2014)	*Ping Gan formula*	*Gastrodia elata*, *Scolopendra subspinipes mutilans*, *Paeoniae lactiflorae*, *Buthus martensii Karsch*, *Arisaematis rhizoma preparatum*, *Radix Angelicae sinensis*, *Radix Glycyrrhizae*, *Semen Zizyphi spinosae*	*Gastrodia elata*:15g *Scolopendra subspinipes mutilans*:5g *Paeoniae lactiflorae*:20g *Buthus martensii Karsch*:8g *Arisaematis rhizoma preparatum*:12g *Radix Angelicae sinensis*: NA *Radix Glycyrrhizae*: NA *Semen Zizyphi spinosae*: NA Decot these herbs with 500cc of water, and take once daily	take once daily/ oral
Liu (2014)	Ping Gan formula	*Gastrodia elata*, *Scolopendra subspinipes mutilans*, *Paeoniae lactiflorae*, *Buthus martensii Karsch*, *Arisaematis rhizoma preparatum*, *Radix Angelicae sinensis*, *Radix Glycyrrhizae*, *Semen Zizyphi spinosae*	*Gastrodia elata*:15g *Scolopendra subspinipes mutilans*:5g *Paeoniae lactiflorae*:20g *Buthus martensii Karsch*:8g *Arisaematis rhizoma preparatum*:12g *Radix Angelicae sinensis*: NA *Radix Glycyrrhizae*: NA *Semen Zizyphi spinosae*: NA Decot these herbs with 500cc of water, and take once daily	take once daily / oral
Lu (2016)	Ping Gan formula	*Gastrodia elata*, *Scolopendra subspinipes mutilans*, *Paeoniae lactiflorae*, *Buthus martensii Karsch*, *Arisaematis rhizoma preparatum*, *Radix Angelicae sinensis*, *Radix Glycyrrhizae*, *Semen Zizyphi spinosae*	*Gastrodia elata*:15g *Scolopendra subspinipes mutilans*:5g *Paeoniae lactiflorae*:20g *Buthus martensii Karsch*:8g *Arisaematis rhizoma preparatum*:12g *Radix Angelicae sinensis*: NA *Radix Glycyrrhizae*: NA *Semen Zizyphi spinosae*: NA Decot these herbs with 500cc of water, and take once daily	take once daily/ oral
Lu (2018)	Ding Xian Tang	*Gastrodia elata*, *Bupleurum chinense *DC., *Scutellaria baicalensis *Georgi, *Magnetitum*, *Dryobalanops aromatica*, *Citri Reticulatae Pericarpium*, *Radix Salviae liguliobae*, *Bulbus fritillariae*, *Cinnabaris*, *Ophiopogon japonicus*, *Radix Glycyrrhizae*, *Arisaematis rhizoma preparatum*	NA	take once every other day / oral
Ma (2001)	Xi Feng Capsules	*Placenta Hominis*, *Gastrodia elata*, *Buthus martensii Karsch*, *Bombyx batryticatus*, *Rhizoma Pinelliae*, *Acori graminei Rhizoma*, *Curcuma aromatica Salisb*.	NA	take twice daily / oral
Su (2019)	Xi Feng Capsules	*Placenta Hominis*, *Gastrodia elata*, *Buthus martensii Karsch*, *Bombyx batryticatus*, *Rhizoma Pinelliae*, *Acori graminei Rhizoma*, *Curcuma aromatica Salisb*., *Alumen*	NA	take twice daily / oral
Xiong (2003)	Xi Feng Capsules	*Cornus officinalis*, *Placenta Hominis*, *Poria cocos*, *Acori graminei Rhizoma*, *Gastrodia elata*, *Buthus martensii Karsch*, *Haematitum*, *Notopterygium incisum*	NA	take two times daily / oral
Yang (2013)	Ping Gan formula	*Gastrodia elata*, *Scolopendra subspinipes mutilans*, *Paeoniae lactiflorae*, *Buthus martensii Karsch*, *Arisaematis rhizoma preparatum*, *Radix Angelicae sinensis*, *Radix Glycyrrhizae*, *Semen Zizyphi spinosae*	*Gastrodia elata*:15g *Scolopendra subspinipes mutilans*:5g *Paeoniae lactiflorae*:20g *Buthus martensii Karsch*:8g *Arisaematis rhizoma preparatum*:12g *Radix Angelicae sinensis*: 20g *Radix Glycyrrhizae*: 8g *Semen Zizyphi spinosae*: 30g It did not mention how to decot these herbs or how often they were taken.	take once daily / oral
Zhong (2015)	Ding Xian Tang	*Codonopsis pilosula*, *Acori graminei Rhizoma*, *Polygala tenuifolia*, *Atractylodes macrocephala*, *Uncaria rhynchophylla* (Miq) Jack., *Succinum*	*Codonopsis pilosula*:6-9g *Acori graminei Rhizoma*:6-9g *Polygala tenuifolia*: 6-9g *Atractylodes macrocephala*: 6-9g *Uncaria rhynchophylla* (Miq) Jack.: 6-12g *Succinum*:0.5-1g It did not mention how to decot these herbs or how often they were taken.	take once daily / oral

NA: not available

**Table 3:** Detail of acupoint (n = 10).

Author (year)	Acupoint	Depth	Specifications	Retention time (min)
Hsu (2004)	CV15, EX-B8, GV8, PC5, ST40	NA	30G (1, 1.5 inch)	30
Lee (2014)	CV14, GV9, GV14, GV20, HT7 SP6, ST36	30–33 mm	NA	30
Liu (2014)	BL15, BL18, BL20, BL23, CV14, GV14, GV20	30–40 mm	NA	30
Lu (2016)	CV14, GV9, GV14, GV20, HT7 SP6, SPT36	30–33 mm	NA	30
Lu (2018)	EX-B8, EX-HN5, GV20, LR3, ST40	NA	NA	30
Ma (2001)	GB20, GV20, GV26, LR3, PC6, ST36	NA	NA	30
Su (2019)	BL10, GV16, ST9	5–15 mm	NA	30
Xiong (2003)	GB20, GV20, GV26, LR3, PC6, ST36	NA	NA	30
Yang (2013)	BL15, BL18, BL20, BL23, CV14, GV14, GV20	30–40 mm	NA	30
Zhong (2015)	CV15, LR3, LU9, PC5, ST36, ST40	NA	30G (1, 1.5 inch)	30

NA: not available

### 3.3. Risk of bias assessment

**Fig 2:**
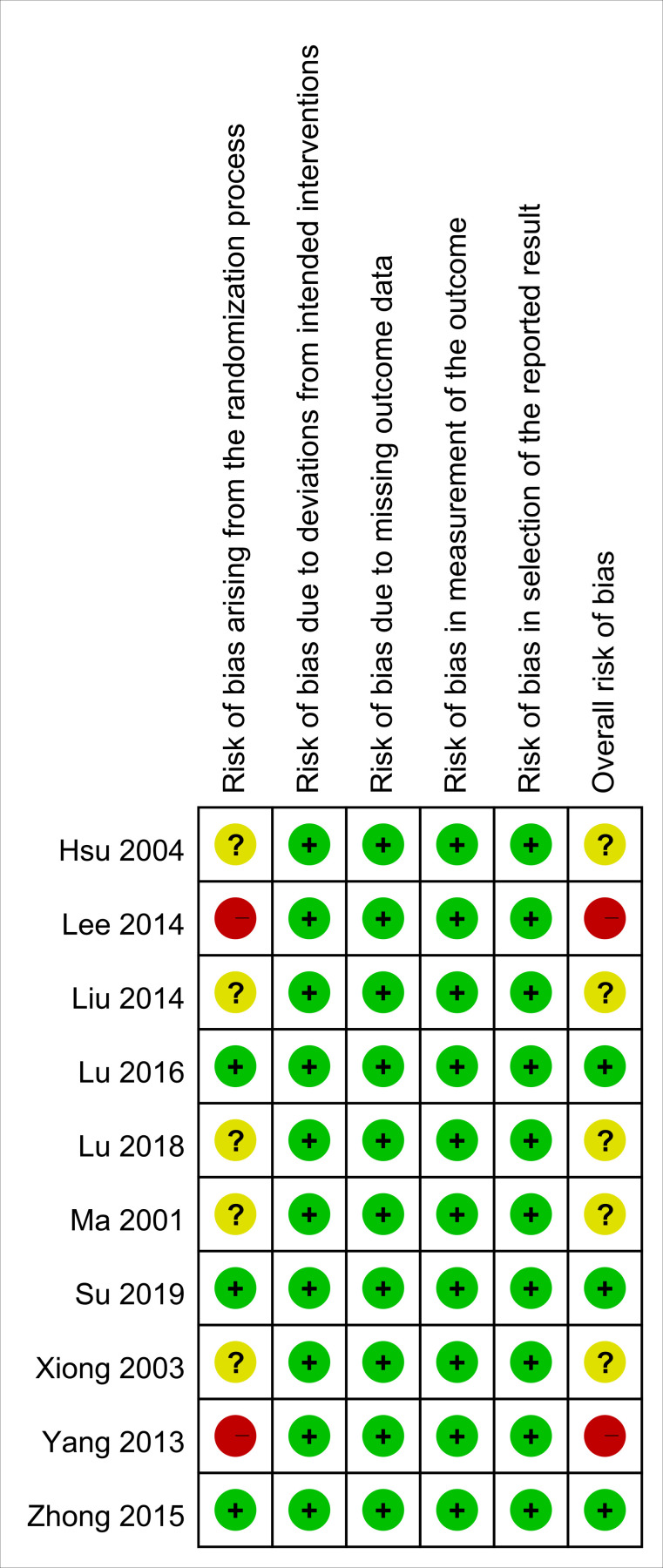
Risk of bias for the included studies.

Fig 2 presents the results of the risk of bias assessment. Among the studies, the risk of bias associated with the randomization process was categorized as “some concern” in five studies [22, 23, 26–28] and “high risk” in two [24, 29], mainly because of the lack of detailed descriptions of the randomization process in these studies. Three studies were categorized as low risk across all the domains. In summary, the overall risk of bias was evaluated as low in three trials [25, 30, 31], unclear in five [22, 23, 26–28], and high in two [24, 29].

### 3.4. Primary and secondary outcomes

#### 3.4.1. Primary outcome: Risk of treatment failure

**Fig 3:**
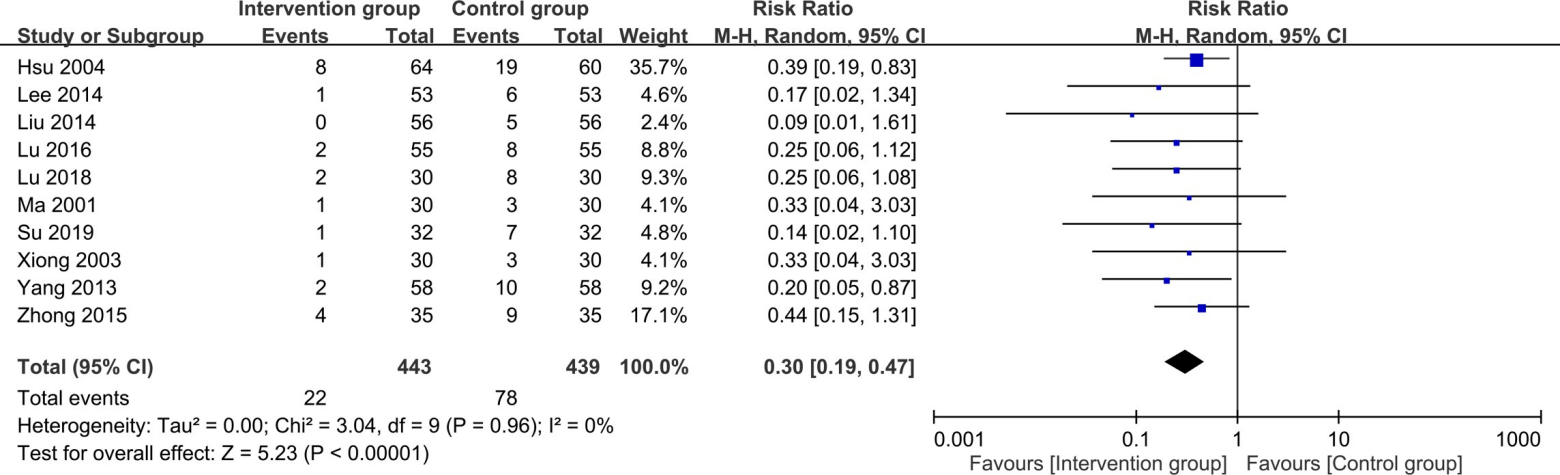
Forest plot presenting the association between herbal medicine-acupuncture combination use (i.e., Intervention group) and the risk of seizure treatment failure. CI: confidence interval. M-H: Mantel–Haenszel.

**Fig 4:**
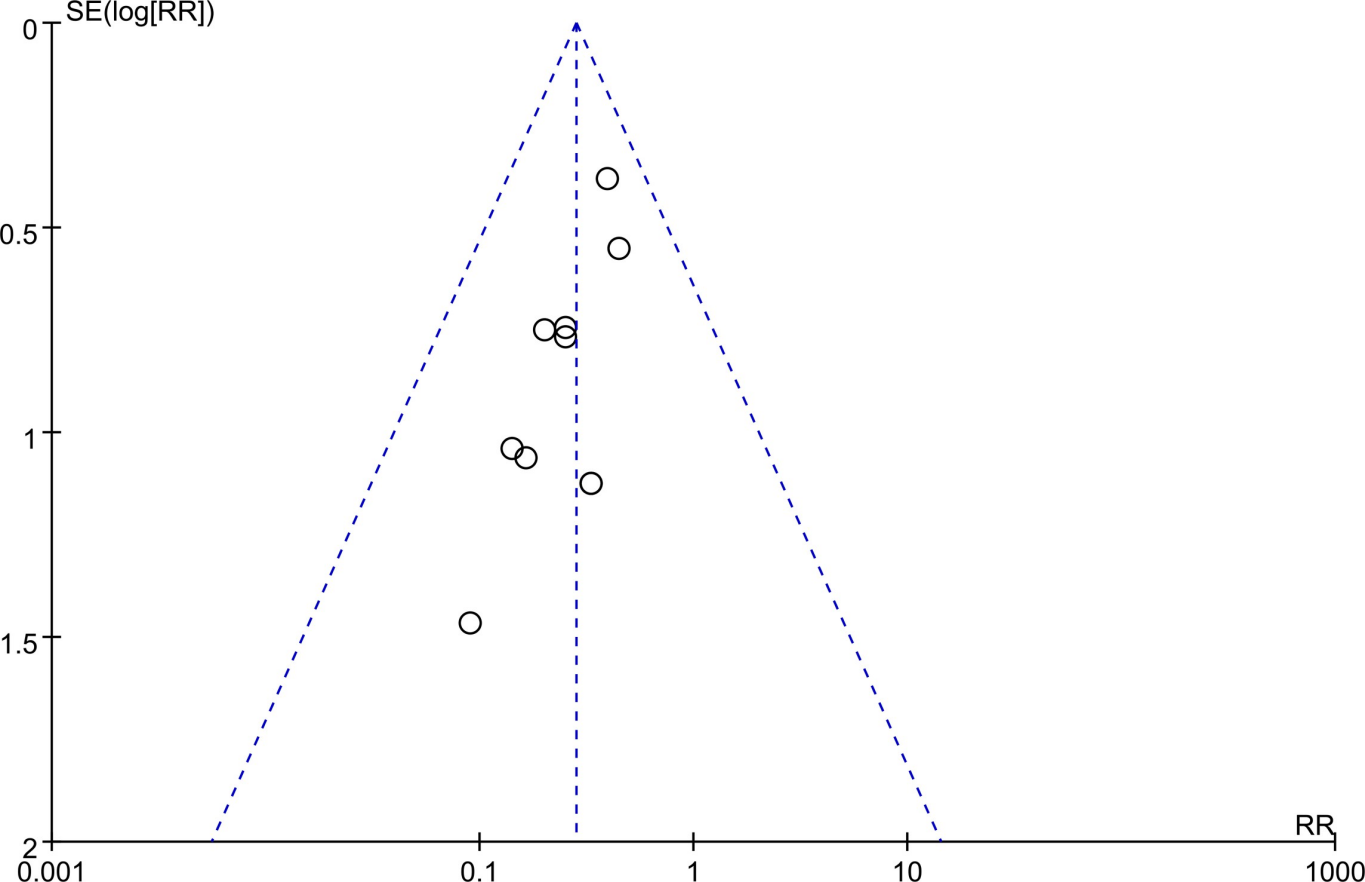
Funnel plot indicating a low risk of publication bias.

**Fig 5:**
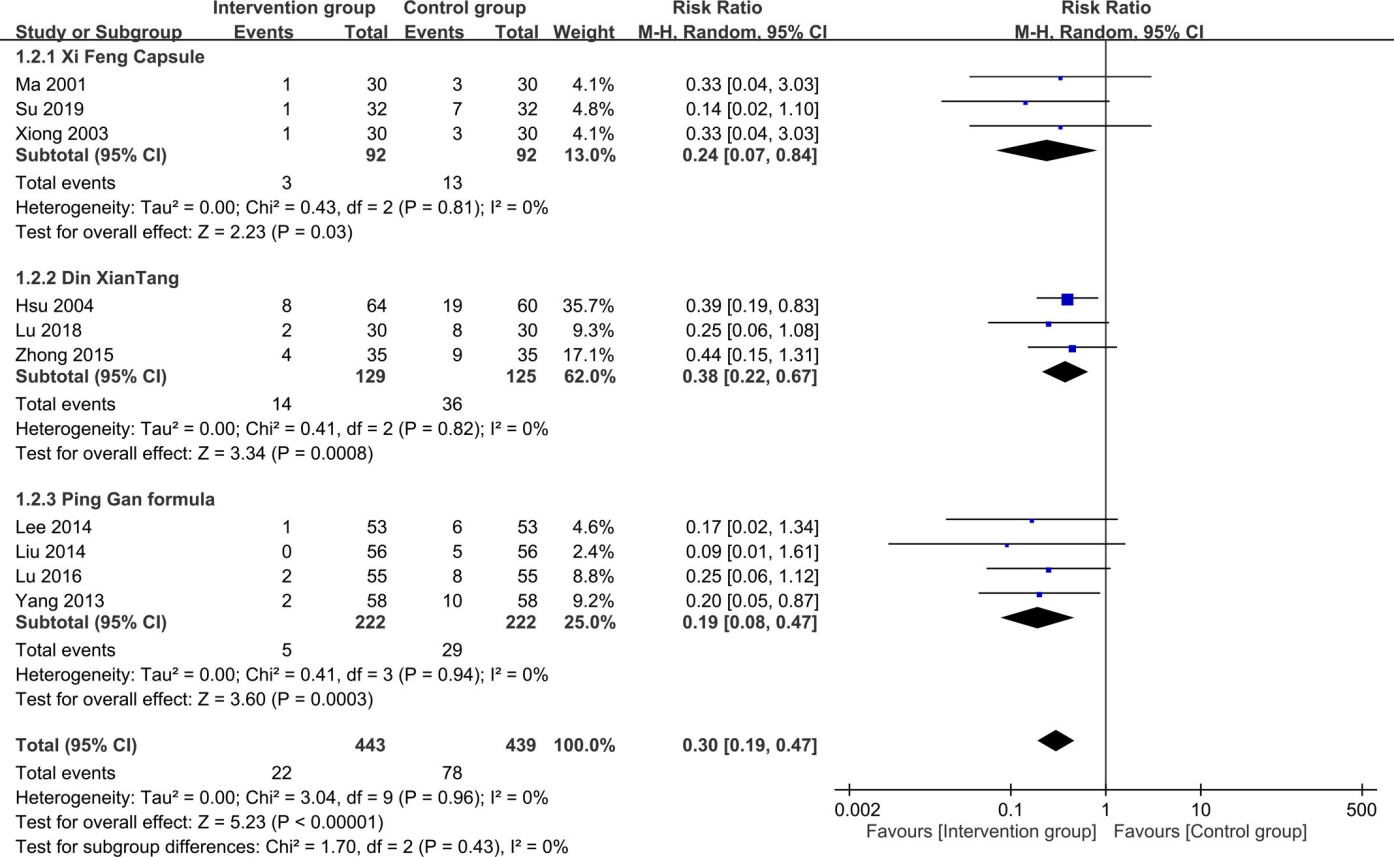
Subgroup analysis based on herbal compound formula. CI: confidence interval. M-H: Mantel–Haenszel.

**Fig 6:**
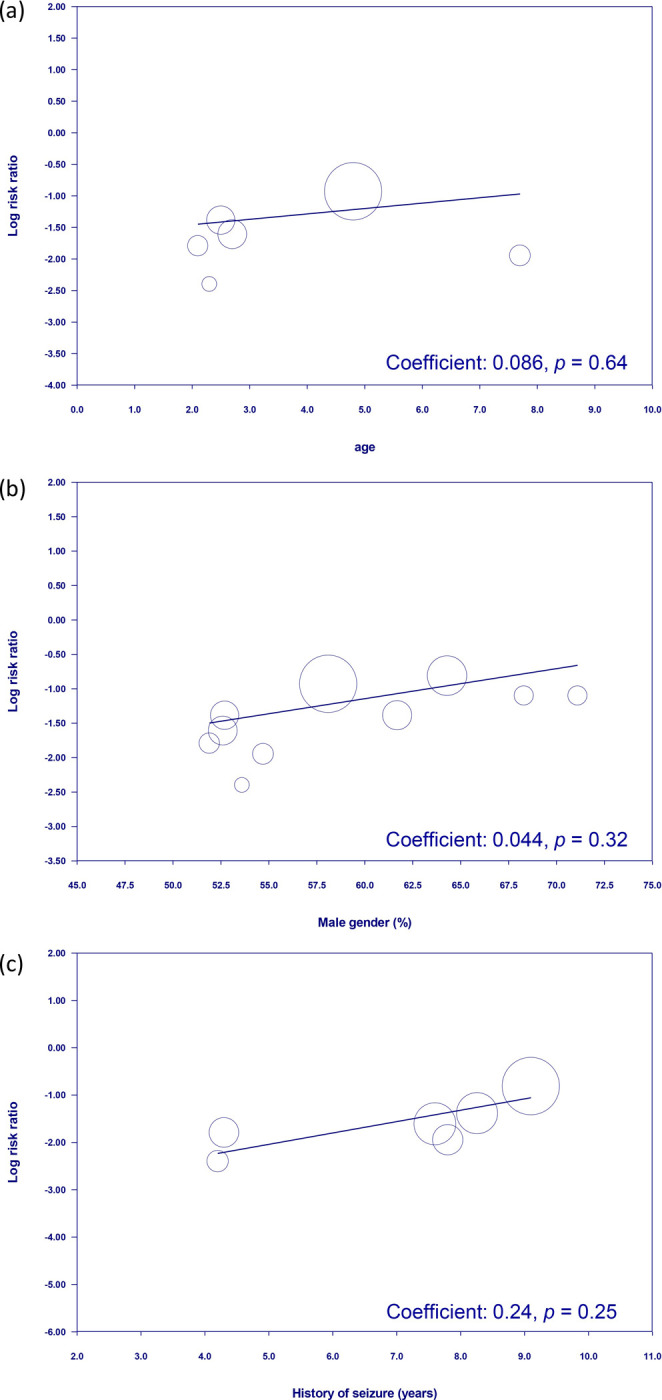
Meta-regression analyses of treatment effects relative to patient demographics and clinical history. The therapeutic efficacy of combined use of herbal medicine and acupuncture for pediatric epilepsy is independent of (a) patient age, (b) male sex, and (c) duration of seizure history.

To assess the risk of treatment failure, the analysis encompassed data from 882 participants (intervention group, n = 443; control group, n = 439). The meta-analysis revealed that patients who received herbal medicine-acupuncture combination had a significantly reduced risk of treatment failure compared with patients who received standard care (RR = 0.3, 95% CI: 0.19–0.47, *P* < 0.00001, I2 = 0%) (Fig 3) [22–31]. This outcome was consistent with that of the sensitivity analysis. Further evaluation of the funnel plot showed minimal publication bias for these outcomes, confirming the representativeness of the studies included in the meta-analysis (Fig 4). The results of the subgroup analysis confirmed the efficacy of herbal medicine-acupuncture combination in managing seizures independent of the specific herbal compound formula (Fig 5). Meta-regression analysis revealed that the beneficial effect of herbal medicine-acupuncture combination was not influenced by the patient age (Fig 6A), male sex (Fig 6B), and duration of seizure history (Fig 6C).

**Fig 7:**
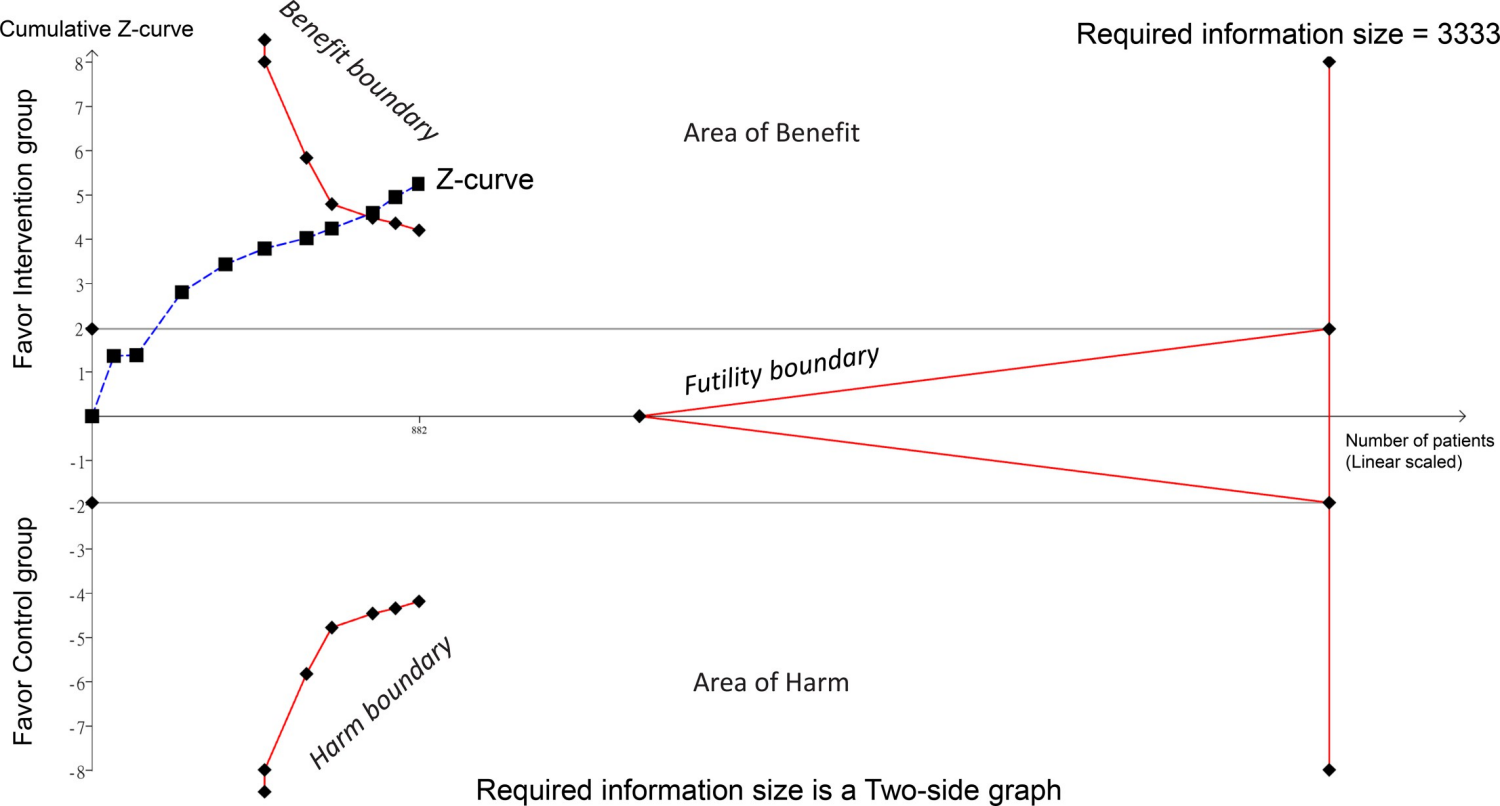
This figure presents the results of the trial sequential analysis (TSA) conducted to evaluate the cumulative evidence. The z-curve representing the cumulative z-score of the data was plotted against the number of participants/events. The TSA-adjusted confidence (i.e., benefit boundary), futility, and traditional confidence (gray) boundaries are also presented. TSA-adjusted boundary: This boundary accounts for repetitive testing on accumulating data and helps maintain an overall 5% risk of type I error. The area inside the boundary indicates the statistically significant zone. Traditional confidence boundary (gray line): This line indicates the conventional boundary for statistical significance without adjustment for repetitive testing. Futility boundary: This optional boundary indicates the point at which the accumulation of more data is unlikely to alter the conclusion of no effect. This is used to determine when further trials are unnecessary.

In the TSA, the z-curve crossed the TSA-adjusted boundary (i.e., the benefit boundary), suggesting that the cumulative evidence has reached a level of statistical significance that is unlikely to be altered by the results of additional trials (Fig 7). This suggests a robust and conclusive effect of herbal medicine-acupuncture combination on seizure treatment.

#### 3.4.2. Secondary outcomes: Impact of herbal medicine/acupuncture on the abnormal rate of EEG and risk of adverse events

**Fig 8:**
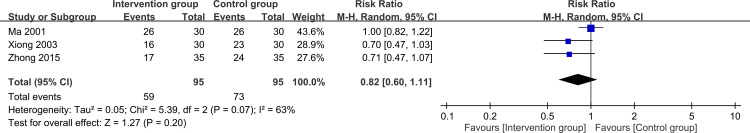
Forest plot presenting the association between herbal medicine-acupuncture combination use (i.e., Intervention group) and the frequency of abnormal EEG. CI: confidence interval. M-H: Mantel–Haenszel.

**Fig 9:**
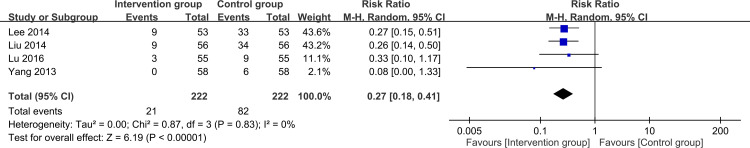
Forest plot showing the association between herbal medicine-acupuncture combination use (i.e., Intervention group) and the risk of adverse events. CI: confidence interval. M-H: Mantel–Haenszel.

Fig 8 presents the effect of herbal medicine-acupuncture combination on EEG recovery. No significant difference was observed in the risk of post-treatment EEG abnormalities (RR = 0.82, 95% CI: 0.6–1.11, *P* = 0.2, *I*^*2*^ = 63%) [27, 28, 30]; however, there appeared to be a nonsignificant trend toward a reduced risk of post-treatment EEG abnormalities among children who received herbal medicine-acupuncture combination (Fig 8). Nevertheless, the application of herbal medicine-acupuncture combination was associated with a lower risk of adverse events compared with the control group (RR = 0.27, 95% CI: 0.18–0.41, *P* < 0.00001, *I*^*2*^ = 0%) (Fig 9) [23–25, 29].

### 3.5. Certainty of evidence

The certainty of evidence for both the risk of treatment failure and risk of adverse events is moderate and has been downgraded owing to concerns related to a high risk of bias in some studies (Table 4). The certainty of evidence for the risk of post-treatment EEG abnormalities is very low, mainly due to the risk of bias, wide CIs, and significant heterogeneity among the study results (Table 4).

**Table 4:** Certainty of evidence.

Outcomes			Domains			Relative effect (95% CI)	No. of participants (studies)	Certainty of the evidence (GRADE)
	A	B	C	D	E			
Risk of treatment failure	**S**	**NS**	**NS**	**NS**	**NS**	**RR 0.3** (0.19 to 0.47)	882 (10 RCTs)	⨁⨁⨁◯ moderate
Risk of abnormal EEG	**S**	**S**	**NS**	**S**	**NS**	**RR 0.82** (0.6 to 1.11)	190 (3 RCTs)	⨁◯◯◯ very low
Risk of adverse events	**S**	**NS**	**NS**	**NS**	**NS**	**RR 0.27** (0.18 to 0.41)	444 (4 RCTs)	⨁⨁⨁◯ moderate

A: risk of bias; B: inconsistency; C: indirectness; D: imprecision; E: potential publication bias; NS: not serious; S: serious; RCT: randomized controlled trial; EEG: electroencephalogram

## 4. Discussion

In this meta-analysis, data of 10 studies that included 882 patients were synthesized. The results indicated that the combined use of herbal medicine and acupuncture exerted therapeutic efficacy for pediatric epilepsy. This combination was associated with a reduced likelihood of treatment failure. Notably, subgroup analyses revealed that certain herbal compound formulas, specifically Ding Xian Tang, Ping Gan, and Xi Feng, were consistently effective than conventional medications in reducing treatment failure rates. Furthermore, the incidence of adverse events was lower in the intervention group than in the control group. While post-treatment EEG abnormalities did not significantly differ between the groups, a significant trend toward improved EEG outcomes was observed in the intervention group.

The occurrence of pediatric epilepsy, characterized by recurrent unprovoked seizures [32], is often indicated by abrupt behavioral shifts such as loss of consciousness and sensory or motor disruptions[2]. The impact of epilepsy transcends health concerns; it can create stigma or discrimination and is associated with greater injury risk and mortality [33]. Although AEDs are considered as standard treatments, their success is limited—only approximately 50% of patients remain seizure-free for 1 year or more after initial treatment with AEDs [34]. This drawback necessitates consideration of additional complementary treatments for children with epilepsy. In China, herbal medicine is widely used in managing epilepsy [35], with research noting that about 27.5% of caregivers have chosen TCM for children with epilepsy [36]. Nevertheless, the efficacy and safety of herbal medicine in the pediatric context remain insufficiently investigated. However, emerging studies highlighted the antiepileptic potential of certain herbal medicines and formulas, hinting at a promising avenue for pediatric care [36–38].

The included studies did not consistently define or quantify the efficacy to allow the extraction of a uniform endpoint across trials. As such, we established our own composite definition of treatment failure based on the following indicators that could be captured from the available data: less than 50% seizure reduction, no improvement or worsening of symptoms, discontinuation due to side effects, and persistently high seizure frequency without improvement per EEG. Despite the variety of these definitions, the heterogeneity in the primary outcome was zero, indicating that our definition may be clinically feasible. Our research demonstrates the therapeutic efficacy of the combined use of herbal medicine and acupuncture for pediatric epilepsy. The robustness of the results of this study is further supported by TSA findings. This aligns with the results of previous studies that reported favorable outcomes from the combined use of herbal medicine and acupuncture for epilepsy in both children and adults [19, 39–41]. Notably, an updated meta-analysis demonstrated the use of herbal medicine as a promising clinical strategy for epilepsy across age groups [19]. Another comprehensive review of 17 RCTs that included participants aged between 1 and 66 years highlighted the potential of herbal medicine to improve seizure control and normalize EEG patterns [38]. Furthermore, a specific review delved into the molecular mechanisms of herbal medicine in pediatric epilepsy treatment, demonstrating its multifaceted approach in regulating neurotransmitters, ion channels, inflammation, apoptosis-related genes, and oxidative stress [39]. Despite this array of evidence, a gap exists in research specifically focused on pediatric patients with unique pharmacokinetic and pharmacodynamic profiles compared with adults [42]. Given these differences, it is imperative to direct our investigative efforts toward understanding how children uniquely respond to herbal medicine-acupuncture combination, ensuring tailored and effective epilepsy treatments for this vulnerable population.

Due to the diverse nature of herbal medicines preparations, outcomes may vary across studies. In the analyzed studies, 3 herbal compound formulas and 22 distinctive single-herb prescriptions were identified (Table 2). However, the main active ingredients within these herbal medicine prescriptions and their specific mechanisms of action in epilepsy treatment have yet to be determined, highlighting the need for further scientific exploration. To investigate the antiepileptic potential of herbal medicine in pediatric care, a subgroup analysis was conducted with a focus on distinct herbal compound formulas, namely, Ping Gan, Ding Xian Tang, and Xi Feng. Although each subgroup comprised a limited number of studies, our findings indicated a decreased risk of seizure treatment failure with these herbal medicines compared with conventional Western medical practices. The Ping Gan formula, traditionally used to “calm the liver yang” and alleviate spasms and convulsions, was the most frequently prescribed herbal medicine in our patient sample [23–25, 29]. Ding Xian Tang is known to be “the most extensively studied antiepileptic herbal medicine formula” [39, 43]. It demonstrated antiseizure and convulsant mitigation properties, particularly in combination with valproic acid, in animal models of pentylenetetrazol-induced chronic epilepsy [44, 45]. Similarly, the Xi Feng capsule has exerted auspicious neuroprotective and antiepileptic effects [46], with its impact on epilepsy treatment appearing to be dose-dependent [47]. In the intricate landscape of herbal medicine, the specific composition of each formula significantly contributes to their therapeutic efficacy. Further research is warranted to establish the optimal therapeutic role of these formulas and to enhance our understanding of their efficacy within pediatric treatment regimens.

Several animal and clinical studies suggest that the combined use of acupuncture and herbal medicine may offer enhanced benefits in treating various diseases, compared to the use of either method alone. In animal studies focusing on polycystic ovary syndrome, liver fibrosis, colitis, intracerebral hemorrhage, and Parkinson’s disease, the combined approach seems to improve treatment effectiveness and outcomes more than each therapy alone [48–52]. In clinical studies, the synergistic application of acupuncture and herbal medicine has significantly enhanced outcomes for conditions such as tic syndrome and cerebral palsy by improving both physical and cognitive function [16, 17]. While the exact mechanisms of how acupuncture and herbal medicine work together are not fully understood, this emerging evidence supports further investigation of these therapies as combined treatments. Our research contributes to increasing evidence that acupuncture and herbal medicine, when used together, can be effective in a variety of clinical situations and patient groups.

Our research demonstrates the safety of herbal medicine and acupuncture in the management of pediatric epilepsy, suggesting reduced incidence of adverse effects compared with conventional AEDs. A significant concern with AEDs is their propensity to cause adverse drug reactions in children, which commonly leads to treatment discontinuation [53, 54]. Notably, adverse drug reactions contribute to as much as 25% of cases of AED treatment discontinuation [55]. Furthermore, detailed systematic review and network meta-analysis have highlighted the potential neurodevelopmental risks associated with valproate, whether used as monotherapy or in combination with other AEDs [56]. This context is prompting a growing shift toward complementary treatments such as herbal medicine and acupuncture, which can be integrated with Western medical practices [36, 57]. Our findings suggest that patients with pediatric epilepsy could benefit from the lower side effect profile of such treatments. However, it is important to continue research into the long-term safety and efficacy of these alternative treatments so as to fully establish their role in pediatric epilepsy treatment.

This meta-analysis has several limitations that need to be acknowledged. First, the methodological quality of the analyzed studies raises concerns, with particular deficiencies in randomization, allocation concealment, and blinding, which could introduce bias into the results. Second, the control of seizures, a key goal in epilepsy treatment, was not consistently monitored across studies due to short follow-up periods. Consequently, the long-term efficacy and recurrence of seizures may not have been sufficiently assessed. Third, these studies frequently overlooked vital outcomes such as reduced epilepsy duration and cognitive side effects, which are essential for a comprehensive evaluation of treatment efficacy and safety. Fourth, the safety profile of herbal medicine-acupuncture combination, while seemingly favorable, is derived from studies with a small sample size, limiting our ability to confirm safety for a wider population. Fifth, our research was mainly based on studies in Chinese language, and the scarcity of large-scale RCTs in our analysis could restrict the broader applicability of our conclusions. Finally, due to the lack of a uniform definition of the efficacy of epilepsy interventions among the trials, we created a composite definition of failure, drawing from the indicators available for data extraction from these trials. The inconsistency in the endpoints used to evaluate the efficacy of epilepsy interventions might undermine the strength of the arguments presented in this manuscript. Given these limitations, the results of this meta-analysis should be interpreted with caution. Further analysis of large-scale, methodologically sound studies is warranted to provide more definitive evidence on the efficacy and safety of herbal medicine and acupuncture in pediatric epilepsy treatment.

## 5. Conclusion

Our meta-analysis demonstrated that herbal medicine and acupuncture could be viable options for pediatric epilepsy treatment. However, the evidence supporting the efficacy of these interventions in the patient population requires further validation. Despite our comprehensive subgroup analyses, there remain additional variables that were not fully explored. The establishment of herbal medicine and acupuncture as a recognized epilepsy treatment will depend on the execution of methodologically robust, multicenter, double-blind RCTs. The use of such stringent research designs is necessary to minimize bias and accurately assess the antiseizure effects of herbal medicine and acupuncture.

## Supporting information

S1 Table Search strategies for databases (results of the primary search conducted on October 20, 2023). (DOCX)

S1 Checklist (DOCX)
