# Supplementary material for: Efficacy and safety of herbal medicine combined with acupuncture in pediatric epilepsy treatment: A meta-analysis of randomized controlled trials
Source: PLoS One. 2024 May 9;19(5):e0303201. doi: 10.1371/journal.pone.0303201 (PMC11081325; doi:10.1371/journal.pone.0303201)
Supplement: S1 Table — (DOCX) [file pone.0303201.s001.docx]

**Supplementary Table 1:** Search strategies for databases (results of the primary search conducted on October 20, 2023)

| **Medline** | | | | | |  |
| --- | --- | --- | --- | --- | --- | --- |
|  | **Command** | **Strategies and keywords** | | **Results** | |  |
| #1 |  | **Search: Traditional Chinese medicine "medicine, chinese traditional"[MeSH Terms] OR ("medicine"[All Fields] AND "chinese"[All Fields] AND "traditional"[All Fields]) OR "chinese traditional medicine"[All Fields] OR ("traditional"[All Fields] AND "chinese"[All Fields] AND "medicine"[All Fields]) OR "traditional chinese medicine"[All Fields]** | | 142,216 | |  |
| #2 |  | **Search: Chinese herbal medicine ("chineses"[All Fields] OR "east asian people"[MeSH Terms] OR ("east"[All Fields] AND "asian"[All Fields] AND "people"[All Fields]) OR "east asian people"[All Fields] OR "chinese"[All Fields]) AND ("plant extracts"[MeSH Terms] OR ("plant"[All Fields] AND "extracts"[All Fields]) OR "plant extracts"[All Fields] OR ("herbal"[All Fields] AND "medicine"[All Fields]) OR "herbal medicine"[All Fields] OR "herbal medicine"[MeSH Terms])** | | 77,977 | |  |
| #3 |  | **Search: Herbal medicine "plant extracts"[MeSH Terms] OR ("plant"[All Fields] AND "extracts"[All Fields]) OR "plant extracts"[All Fields] OR ("herbal"[All Fields] AND "medicine"[All Fields]) OR "herbal medicine"[All Fields] OR "herbal medicine"[MeSH Terms] virus 2"[Title/Abstract])) OR ("severe acute respiratory syndrome coronavirus 2"[Title/Abstract]))** | | 261,885 | |  |
| #4 |  | **Search: herbal "herbal medicine"[MeSH Terms] OR ("herbal"[All Fields] AND "medicine"[All Fields]) OR "herbal medicine"[All Fields] OR "herbalism"[All Fields] OR "herbal"[All Fields] OR "herbals"[All Fields]** | | 102,770 | |  |
| #5 |  | **Search: herb "herb"[All Fields]** | | 24,014 | |  |
| #6 |  | **Search:** botanical "botanic"[All Fields] OR "botanical"[All Fields] OR "botanically"[All Fields] OR "botanicals"[All Fields] OR "botanics"[All Fields] | | 32,421 | |  |
| #7 | #1 OR #2 OR #3 OR #4 OR #5 OR #6 | **Search: "medicine, chinese traditional"[MeSH Terms] OR ("medicine"[All Fields] AND "chinese"[All Fields] AND "traditional"[All Fields]) OR "chinese traditional medicine"[All Fields] OR ("traditional"[All Fields] AND "chinese"[All Fields] AND "medicine"[All Fields]) OR "traditional chinese medicine"[All Fields] OR (("chineses"[All Fields] OR "east asian people"[MeSH Terms] OR ("east"[All Fields] AND "asian"[All Fields] AND "people"[All Fields]) OR "east asian people"[All Fields] OR "chinese"[All Fields]) AND ("plant extracts"[MeSH Terms] OR ("plant"[All Fields] AND "extracts"[All Fields]) OR "plant extracts"[All Fields] OR ("herbal"[All Fields] AND "medicine"[All Fields]) OR "herbal medicine"[All Fields] OR "herbal medicine"[MeSH Terms])) OR ("plant extracts"[MeSH Terms] OR ("plant"[All Fields] AND "extracts"[All Fields]) OR "plant extracts"[All Fields] OR ("herbal"[All Fields] AND "medicine"[All Fields]) OR "herbal medicine"[All Fields] OR "herbal medicine"[MeSH Terms]) OR ("herbal medicine"[MeSH Terms] OR ("herbal"[All Fields] AND "medicine"[All Fields]) OR "herbal medicine"[All Fields] OR "herbalism"[All Fields] OR "herbal"[All Fields] OR "herbals"[All Fields]) OR "herb"[All Fields] OR ("botanic"[All Fields] OR "botanical"[All Fields] OR "botanically"[All Fields] OR "botanicals"[All Fields] OR "botanics"[All Fields])** | | 416,395 | |  |
| #8 |  | **Search: Acupuncture "acupunctural"[All Fields] OR "acupuncture"[MeSH Terms] OR "acupuncture"[All Fields] OR "acupuncture therapy"[MeSH Terms] OR ("acupuncture"[All Fields] AND "therapy"[All Fields]) OR "acupuncture therapy"[All Fields] OR "acupuncture s"[All Fields] OR "acupunctured"[All Fields] OR "acupunctures"[All Fields] OR "acupuncturing"[All Fields]** | | 43,447 | |  |
| #9 |  | **Search: a**cupuncture therapy "acupuncture therapy"[MeSH Terms] OR ("acupuncture"[All Fields] AND "therapy"[All Fields]) OR "acupuncture therapy"[All Fields] | | 35,679 | |  |
| #10 |  | **Search:** needle acupuncture ("needle s"[All Fields] OR "needled"[All Fields] OR "needles"[MeSH Terms] OR "needles"[All Fields] OR "needle"[All Fields] OR "needlings"[All Fields] OR "percutaneous collagen induction"[MeSH Terms] OR ("percutaneous"[All Fields] AND "collagen"[All Fields] AND "induction"[All Fields]) OR "percutaneous collagen induction"[All Fields] OR "needling"[All Fields]) AND ("acupunctural"[All Fields] OR "acupuncture"[MeSH Terms] OR "acupuncture"[All Fields] OR "acupuncture therapy"[MeSH Terms] OR ("acupuncture"[All Fields] AND "therapy"[All Fields]) OR "acupuncture therapy"[All Fields] OR "acupuncture s"[All Fields] OR "acupunctured"[All Fields] OR "acupunctures"[All Fields] OR "acupuncturing"[All Fields]) | | 5,703 | |  |
| #11 |  | **Search:** auricular acupuncture "acupuncture, ear"[MeSH Terms] OR ("acupuncture"[All Fields] AND "ear"[All Fields]) OR "ear acupuncture"[All Fields] OR ("auricular"[All Fields] AND "acupuncture"[All Fields]) OR "auricular acupuncture"[All Fields] | | 1,787 | |  |
| #12 | #8 OR #9 OR #10 OR #11 | **Search:** "acupunctural"[All Fields] OR "acupuncture"[MeSH Terms] OR "acupuncture"[All Fields] OR "acupuncture therapy"[MeSH Terms] OR ("acupuncture"[All Fields] AND "therapy"[All Fields]) OR "acupuncture therapy"[All Fields] OR "acupuncture s"[All Fields] OR "acupunctured"[All Fields] OR "acupunctures"[All Fields] OR "acupuncturing"[All Fields] OR ("acupuncture therapy"[MeSH Terms] OR ("acupuncture"[All Fields] AND "therapy"[All Fields]) OR "acupuncture therapy"[All Fields]) OR (("needle s"[All Fields] OR "needled"[All Fields] OR "needles"[MeSH Terms] OR "needles"[All Fields] OR "needle"[All Fields] OR "needlings"[All Fields] OR "percutaneous collagen induction"[MeSH Terms] OR ("percutaneous"[All Fields] AND "collagen"[All Fields] AND "induction"[All Fields]) OR "percutaneous collagen induction"[All Fields] OR "needling"[All Fields]) AND ("acupunctural"[All Fields] OR "acupuncture"[MeSH Terms] OR "acupuncture"[All Fields] OR "acupuncture therapy"[MeSH Terms] OR ("acupuncture"[All Fields] AND "therapy"[All Fields]) OR "acupuncture therapy"[All Fields] OR "acupuncture s"[All Fields] OR "acupunctured"[All Fields] OR "acupunctures"[All Fields] OR "acupuncturing"[All Fields])) OR ("acupuncture, ear"[MeSH Terms] OR ("acupuncture"[All Fields] AND "ear"[All Fields]) OR "ear acupuncture"[All Fields] OR ("auricular"[All Fields] AND "acupuncture"[All Fields]) OR "auricular acupuncture"[All Fields]) | | 43,447 | |  |
| #13 |  | **Search:** epilepsy "epilepsie"[All Fields] OR "epilepsy"[MeSH Terms] OR "epilepsy"[All Fields] OR "epilepsies"[All Fields] OR "epilepsy s"[All Fields] | | 182,018 | |  |
| #14 |  | **Search:** pediatrics "paediatrics"[All Fields] OR "pediatrics"[MeSH Terms] OR "pediatrics"[All Fields] OR "paediatric"[All Fields] OR "pediatric"[All Fields] | | 1,246,886 | |  |
| #15 | #7 AND #12 AND #13 AND #14 | **Search:** ("medicine, chinese traditional"[MeSH Terms] OR ("medicine"[All Fields] AND "chinese"[All Fields] AND "traditional"[All Fields]) OR "chinese traditional medicine"[All Fields] OR ("traditional"[All Fields] AND "chinese"[All Fields] AND "medicine"[All Fields]) OR "traditional chinese medicine"[All Fields] OR (("chineses"[All Fields] OR "east asian people"[MeSH Terms] OR ("east"[All Fields] AND "asian"[All Fields] AND "people"[All Fields]) OR "east asian people"[All Fields] OR "chinese"[All Fields]) AND ("plant extracts"[MeSH Terms] OR ("plant"[All Fields] AND "extracts"[All Fields]) OR "plant extracts"[All Fields] OR ("herbal"[All Fields] AND "medicine"[All Fields]) OR "herbal medicine"[All Fields] OR "herbal medicine"[MeSH Terms])) OR ("plant extracts"[MeSH Terms] OR ("plant"[All Fields] AND "extracts"[All Fields]) OR "plant extracts"[All Fields] OR ("herbal"[All Fields] AND "medicine"[All Fields]) OR "herbal medicine"[All Fields] OR "herbal medicine"[MeSH Terms]) OR ("herbal medicine"[MeSH Terms] OR ("herbal"[All Fields] AND "medicine"[All Fields]) OR "herbal medicine"[All Fields] OR "herbalism"[All Fields] OR "herbal"[All Fields] OR "herbals"[All Fields]) OR "herb"[All Fields] OR ("botanic"[All Fields] OR "botanical"[All Fields] OR "botanically"[All Fields] OR "botanicals"[All Fields] OR "botanics"[All Fields])) AND ("acupunctural"[All Fields] OR "acupuncture"[MeSH Terms] OR "acupuncture"[All Fields] OR "acupuncture therapy"[MeSH Terms] OR ("acupuncture"[All Fields] AND "therapy"[All Fields]) OR "acupuncture therapy"[All Fields] OR "acupuncture s"[All Fields] OR "acupunctured"[All Fields] OR "acupunctures"[All Fields] OR "acupuncturing"[All Fields] OR ("acupuncture therapy"[MeSH Terms] OR ("acupuncture"[All Fields] AND "therapy"[All Fields]) OR "acupuncture therapy"[All Fields]) OR (("needle s"[All Fields] OR "needled"[All Fields] OR "needles"[MeSH Terms] OR "needles"[All Fields] OR "needle"[All Fields] OR "needlings"[All Fields] OR "percutaneous collagen induction"[MeSH Terms] OR ("percutaneous"[All Fields] AND "collagen"[All Fields] AND "induction"[All Fields]) OR "percutaneous collagen induction"[All Fields] OR "needling"[All Fields]) AND ("acupunctural"[All Fields] OR "acupuncture"[MeSH Terms] OR "acupuncture"[All Fields] OR "acupuncture therapy"[MeSH Terms] OR ("acupuncture"[All Fields] AND "therapy"[All Fields]) OR "acupuncture therapy"[All Fields] OR "acupuncture s"[All Fields] OR "acupunctured"[All Fields] OR "acupunctures"[All Fields] OR "acupuncturing"[All Fields])) OR ("acupuncture, ear"[MeSH Terms] OR ("acupuncture"[All Fields] AND "ear"[All Fields]) OR "ear acupuncture"[All Fields] OR ("auricular"[All Fields] AND "acupuncture"[All Fields]) OR "auricular acupuncture"[All Fields])) AND ("epilepsie"[All Fields] OR "epilepsy"[MeSH Terms] OR "epilepsy"[All Fields] OR "epilepsies"[All Fields] OR "epilepsy s"[All Fields]) AND ("paediatrics"[All Fields] OR "pediatrics"[MeSH Terms] OR "pediatrics"[All Fields] OR "paediatric"[All Fields] OR "pediatric"[All Fields]) | | 124 | |  |
|  | Final | ENGLISH | | **(124)** | |  |
| **Embase** | | | | | |  |
| **R** | **Command** | **Strategies and keywords** | **Results** | | |  |
| #1 |  | **Search: Traditional Chinese medicine/** | 45,428 | | |  |
| #2 |  | **Search: Chinese herbal medicine (Chinese medicine)/** | 57,327 | | |  |
| #3 |  | **Search: Herbal medicine/** | 29,591 | | |  |
| #4 |  | **Search: herbal (herbal.mp)/** | 88,905 | | |  |
| #5 |  | **Search: herb "herb"[All Fields]** | 11,468 | | |  |
| #6 |  | **Search:** botanical **(**botanical**.mp)/** | 15,047 | | |  |
| #7 | #1 OR #2 OR #3 OR #4 OR #5 OR #6 | **Search: "medicine, chinese traditional"[MeSH Terms] OR ("medicine"[All Fields] AND "chinese"[All Fields] AND "traditional"[All Fields]) OR "chinese traditional medicine"[All Fields] OR ("traditional"[All Fields] AND "chinese"[All Fields] AND "medicine"[All Fields]) OR "traditional chinese medicine"[All Fields] OR (("chineses"[All Fields] OR "east asian people"[MeSH Terms] OR ("east"[All Fields] AND "asian"[All Fields] AND "people"[All Fields]) OR "east asian people"[All Fields] OR "chinese"[All Fields]) AND ("plant extracts"[MeSH Terms] OR ("plant"[All Fields] AND "extracts"[All Fields]) OR "plant extracts"[All Fields] OR ("herbal"[All Fields] AND "medicine"[All Fields]) OR "herbal medicine"[All Fields] OR "herbal medicine"[MeSH Terms])) OR ("plant extracts"[MeSH Terms] OR ("plant"[All Fields] AND "extracts"[All Fields]) OR "plant extracts"[All Fields] OR ("herbal"[All Fields] AND "medicine"[All Fields]) OR "herbal medicine"[All Fields] OR "herbal medicine"[MeSH Terms]) OR ("herbal medicine"[MeSH Terms] OR ("herbal"[All Fields] AND "medicine"[All Fields]) OR "herbal medicine"[All Fields] OR "herbalism"[All Fields] OR "herbal"[All Fields] OR "herbals"[All Fields]) OR "herb"[All Fields] OR ("botanic"[All Fields] OR "botanical"[All Fields] OR "botanically"[All Fields] OR "botanicals"[All Fields] OR "botanics"[All Fields])** | 172,809 | | |  |
| #8 |  | **Search: Acupuncture/** | 48,224 | | |  |
| #9 |  | **Search: a**cupuncture therapy. **mp/** | 2,509 | | |  |
| #10 |  | **Search:** needle acupuncture.mp. or acupuncture needle/ | 2,759 | | |  |
| #11 |  | **Search:** auricular acupuncture/ | 852 | | |  |
| #12 | #8 OR #9 OR #10 OR #11 | **Search:** "acupunctural"[All Fields] OR "acupuncture"[MeSH Terms] OR "acupuncture"[All Fields] OR "acupuncture therapy"[MeSH Terms] OR ("acupuncture"[All Fields] AND "therapy"[All Fields]) OR "acupuncture therapy"[All Fields] OR "acupuncture s"[All Fields] OR "acupunctured"[All Fields] OR "acupunctures"[All Fields] OR "acupuncturing"[All Fields] OR ("acupuncture therapy"[MeSH Terms] OR ("acupuncture"[All Fields] AND "therapy"[All Fields]) OR "acupuncture therapy"[All Fields]) OR (("needle s"[All Fields] OR "needled"[All Fields] OR "needles"[MeSH Terms] OR "needles"[All Fields] OR "needle"[All Fields] OR "needlings"[All Fields] OR "percutaneous collagen induction"[MeSH Terms] OR ("percutaneous"[All Fields] AND "collagen"[All Fields] AND "induction"[All Fields]) OR "percutaneous collagen induction"[All Fields] OR "needling"[All Fields]) AND ("acupunctural"[All Fields] OR "acupuncture"[MeSH Terms] OR "acupuncture"[All Fields] OR "acupuncture therapy"[MeSH Terms] OR ("acupuncture"[All Fields] AND "therapy"[All Fields]) OR "acupuncture therapy"[All Fields] OR "acupuncture s"[All Fields] OR "acupunctured"[All Fields] OR "acupunctures"[All Fields] OR "acupuncturing"[All Fields])) OR ("acupuncture, ear"[MeSH Terms] OR ("acupuncture"[All Fields] AND "ear"[All Fields]) OR "ear acupuncture"[All Fields] OR ("auricular"[All Fields] AND "acupuncture"[All Fields]) OR "auricular acupuncture"[All Fields]) | 49,900 | | |  |
| #13 |  | **Search:** epilepsy Rolandic epilepsy/ or severe myoclonic epilepsy in infancy/ or mesial temporal lobe epilepsy with hippocampal sclerosis/ or benign occipital epilepsy/ or abdominal epilepsy/ or experimental epilepsy/ or occipital lobe epilepsy/ or lateral temporal lobe epilepsy/ or awakening epilepsy/ or idiopathic epilepsy/ or atypical absence epilepsy/ or musicogenic epilepsy/ or post encephalitic epilepsy/ or post stroke epilepsy/ or onchocerciasis-associated epilepsy/ or typical absence epilepsy/ or mesial temporal lobe epilepsy/ or focal neocortical epilepsy/ or refractory focal epilepsy/ or temporal lobe epilepsy/ or frontal lobe epilepsy/ or epilepsy alarm monitor/ or infantile myoclonic epilepsy/ or reflex epilepsy/ or extratemporal epilepsy/ or juvenile myoclonic epilepsy/ or familial partial epilepsy/ or parietal lobe epilepsy/ or pyridoxine-dependent epilepsy/ or generalized epilepsy/ or hippocampal epilepsy/ or grand mal epilepsy/ or "Quality of Life in Childhood Epilepsy Questionnaire"/ or myoclonic astatic epilepsy/ or benign rolandic epilepsy/ or reading epilepsy/ or "benign focal epilepsy of childhood"/ or epilepsy.mp. or limbic epilepsy/ or "benign myoclonic epilepsy of infancy"/ or late onset epilepsy/ or refractory mesial temporal lobe epilepsy/ or symptomatic generalized epilepsy/ or "seizure, epilepsy and convulsion"/ or autosomal dominant lateral temporal lobe epilepsy/ or idiopathic generalized epilepsy/ or autoimmune epilepsy/ or refractory epilepsy/ or non-convulsive epilepsy/ or progressive myoclonus epilepsy/ or progressive familial myoclonus epilepsy/ or partial motor epilepsy/ or genetic generalized epilepsy/ or sensory partial epilepsy/ or photosensitive epilepsy/ or epilepsy with myoclonic absences/ or nocturnal frontal lobe epilepsy/ or juvenile absence epilepsy/ or myoclonus epilepsy/ or startle epilepsy/ or absence epilepsy/ or catamenial epilepsy/ or nocturnal epilepsy/ or generalized epilepsy with febrile seizures-plus/ or epilepsy/ or traumatic epilepsy/ or benign childhood epilepsy/ or genetically epilepsy prone rat/ or focal epilepsy/ or complex partial epilepsy/ or infantile epilepsy/ or febrile infection related epilepsy syndrome/ or childhood absence epilepsy/ or sudden unexpected death in epilepsy/ or benign adult familial myoclonic epilepsy/ or symptomatic epilepsy/ or childhood epilepsy/ | 256,,808 | | |  |
| #14 |  | **Search:** pediatrics / | 94,280 | | |  |
| #15 | #7 AND #12 AND #13 AND #14 | **Search:** ("medicine, chinese traditional"[MeSH Terms] OR ("medicine"[All Fields] AND "chinese"[All Fields] AND "traditional"[All Fields]) OR "chinese traditional medicine"[All Fields] OR ("traditional"[All Fields] AND "chinese"[All Fields] AND "medicine"[All Fields]) OR "traditional chinese medicine"[All Fields] OR (("chineses"[All Fields] OR "east asian people"[MeSH Terms] OR ("east"[All Fields] AND "asian"[All Fields] AND "people"[All Fields]) OR "east asian people"[All Fields] OR "chinese"[All Fields]) AND ("plant extracts"[MeSH Terms] OR ("plant"[All Fields] AND "extracts"[All Fields]) OR "plant extracts"[All Fields] OR ("herbal"[All Fields] AND "medicine"[All Fields]) OR "herbal medicine"[All Fields] OR "herbal medicine"[MeSH Terms])) OR ("plant extracts"[MeSH Terms] OR ("plant"[All Fields] AND "extracts"[All Fields]) OR "plant extracts"[All Fields] OR ("herbal"[All Fields] AND "medicine"[All Fields]) OR "herbal medicine"[All Fields] OR "herbal medicine"[MeSH Terms]) OR ("herbal medicine"[MeSH Terms] OR ("herbal"[All Fields] AND "medicine"[All Fields]) OR "herbal medicine"[All Fields] OR "herbalism"[All Fields] OR "herbal"[All Fields] OR "herbals"[All Fields]) OR "herb"[All Fields] OR ("botanic"[All Fields] OR "botanical"[All Fields] OR "botanically"[All Fields] OR "botanicals"[All Fields] OR "botanics"[All Fields])) AND ("acupunctural"[All Fields] OR "acupuncture"[MeSH Terms] OR "acupuncture"[All Fields] OR "acupuncture therapy"[MeSH Terms] OR ("acupuncture"[All Fields] AND "therapy"[All Fields]) OR "acupuncture therapy"[All Fields] OR "acupuncture s"[All Fields] OR "acupunctured"[All Fields] OR "acupunctures"[All Fields] OR "acupuncturing"[All Fields] OR ("acupuncture therapy"[MeSH Terms] OR ("acupuncture"[All Fields] AND "therapy"[All Fields]) OR "acupuncture therapy"[All Fields]) OR (("needle s"[All Fields] OR "needled"[All Fields] OR "needles"[MeSH Terms] OR "needles"[All Fields] OR "needle"[All Fields] OR "needlings"[All Fields] OR "percutaneous collagen induction"[MeSH Terms] OR ("percutaneous"[All Fields] AND "collagen"[All Fields] AND "induction"[All Fields]) OR "percutaneous collagen induction"[All Fields] OR "needling"[All Fields]) AND ("acupunctural"[All Fields] OR "acupuncture"[MeSH Terms] OR "acupuncture"[All Fields] OR "acupuncture therapy"[MeSH Terms] OR ("acupuncture"[All Fields] AND "therapy"[All Fields]) OR "acupuncture therapy"[All Fields] OR "acupuncture s"[All Fields] OR "acupunctured"[All Fields] OR "acupunctures"[All Fields] OR "acupuncturing"[All Fields])) OR ("acupuncture, ear"[MeSH Terms] OR ("acupuncture"[All Fields] AND "ear"[All Fields]) OR "ear acupuncture"[All Fields] OR ("auricular"[All Fields] AND "acupuncture"[All Fields]) OR "auricular acupuncture"[All Fields])) AND ("epilepsie"[All Fields] OR "epilepsy"[MeSH Terms] OR "epilepsy"[All Fields] OR "epilepsies"[All Fields] OR "epilepsy s"[All Fields]) AND ("paediatrics"[All Fields] OR "pediatrics"[MeSH Terms] OR "pediatrics"[All Fields] OR "paediatric"[All Fields] OR "pediatric"[All Fields]) | 26 | | |  |
|  | Final | ENGLISH | **(26)** | | |  |
| **Cochrane Library** | | | | | |  |
| **R** | **Command** | **Strategies and keywords** | **Results** | | |  |
| #1 |  | **Search: Traditional Chinese medicine**  Synonyms: Chinese Medicine, Traditional; Chinese Traditional Medicine; Traditional Medicine, Chinese; Traditional Chinese Medicine; Zhong Yi Xue; Chung I Hsueh; Hsueh, Chung I; Traditional Tongue Assessments; Tongue Diagnoses, Traditional; Traditional Tongue Assessment; Tongue Assessment, Traditional; Tongue Diagnosis, Traditional; Traditional Tongue Diagnosis; Traditional Tongue Diagnoses | 10,619 | | |  |
| #2 |  | **Search: Chinese herbal medicine**  Synonyms: Chinese Herbal Drugs; Chinese Drugs, Plant; Herbal Drugs, Chinese; Extracts, Chinese Plant; Chinese Plant Extracts; Plant Extracts, Chinese | 3,620 | | |  |
| #3 |  | **Search: Herbal medicine**  Synonyms: Herbal Medicine Synonyms: Hawaiian Herbal Medicine; Laau Lapaau; Medicine, Hawaiian Herbal; Herbal Medicine, Hawaiian; La'au Lapa'au; La au Lapa au; Herbalism; Medicine, Herbal Plant Extracts Synonyms: Herbal Medicines; Medicines, Herbal; Plant Extract; Extract, Plant; Extracts, Plant | 6,214 | | |  |
| #4 |  | **Search: herbal**  Synonyms: Herbals | 11,614 | | |  |
| #5 |  | **Search: herb**  Phytotherapy Synonyms: Herbal Therapy; Herb Therapy | 2,798 | | |  |
| #6 |  | **Search:** botanical  Synonyms: Botanical Antineoplastics; Antineoplastics, Botanical | 507 | | |  |
| #7 | #1 OR #2 OR #3 OR #4 OR #5 OR #6 |  | 12,059 | | |  |
| #8 |  | **Search: Acupuncture**  Synonyms: Pharmacopuncture | 19,391 | | |  |
| #9 |  | **Search:** acupuncture therapy  Synonyms: Acupuncture Treatment; Treatment, Acupuncture; Acupuncture Treatments; Therapy, Acupuncture; Pharmacoacupuncture Therapy; Pharmacoacupuncture Treatment; Therapy, Pharmacoacupuncture; Treatment, Pharmacoacupuncture; Acupotomies; Acupotomy | 10,789 | | |  |
| #10 |  | **Search:** needle acupuncture  Synonyms: Pharmacopuncture | 3,967 | | |  |
| #11 |  | **Search:** auricular acupuncture  Synonyms: Ear Acupuncture; Auricular Acupuncture; Acupuncture, Auricular; Acupunctures, Auricular; Auricular Acupunctures; Acupunctures, Ear; Ear Acupunctures | 952 | | |  |
| #12 | #8 OR #9 OR #10 OR #11 |  | 19.391 | | |  |
| #13 |  | **Search:** epilepsy  Synonyms: Seizure Disorders; Epilepsies; Seizure Disorder; Cryptogenic Epilepsies; Epilepsies, Cryptogenic; Epilepsy, Cryptogenic; Cryptogenic Epilepsy; Auras; Aura; Epilepsy, Awakening; Awakening Epilepsy | 8,539 | | |  |
| #14 |  | **Search:** pediatrics  Synonyms: Pediatric Psychology; Psychology, Pediatric | 43,823 | | |  |
| #15 | #7 AND #12 AND #13 AND #14 |  | 3 | | |  |
|  | Final | ENGLISH | **(3)** | | |  |
| **China National Knowledge Infrastructure (in Chinese)** | | | | | |  |
| **R** | **Command** | **Strategies and keywords** | | **Results** | |  |
| #1 |  | **Search: Traditional Chinese medicine** (in Chinese) | | 363,494 | |  |
| #2 |  | **Search: Chinese herbal medicine** (in Chinese) | | 472,265 | |  |
| #3 |  | **Search: Herbal medicine** (in Chinese) | | 40,838 | |  |
| #4 |  | **Search: herbal** (in Chinese) | | 3,584 | |  |
| #5 |  | **Search: herb** (in Chinese) | | 3,584 | |  |
| #6 |  | **Search:** botanical (in Chinese) | | 919,691 | |  |
| #7 | #1 OR #2 OR #3 OR #4 OR #5 OR #6 |  | | 1,665,293 | |  |
| #8 |  | **Search: Acupuncture** (in Chinese) | | 121,300 | |  |
| #9 |  | **Search:** acupuncture therapy (in Chinese) | | 1,160 | |  |
| #10 |  | **Search:** needle acupuncture (in Chinese) | | 139,658 | |  |
| #11 |  | **Search:** auricular acupuncture (in Chinese) | | 3,818 | |  |
| #12 | #8 OR #9 OR #10 OR #11 |  | | 223,408 | |  |
| #13 |  | **Search:** epilepsy (in Chinese) | | 64,238 | |  |
| #14 |  | **Search:** pediatrics (in Chinese) | | 263,309 | |  |
| #15 | #7 AND #12 AND #13 AND #14 |  | | 126 | |  |
|  | Final | CHINESE | | **(126)** | |  |
| **CQVIP Database for Chinese Technical Periodicals (in Chinese)** | | | | | | |
| **R** | **Command** | **Strategies and keywords** | | | **Results** | |
| #1 |  | **Search: Traditional Chinese medicine** (in Chinese) | | | 10,224 | |
| #2 |  | **Search: Chinese herbal medicine** (in Chinese) | | | 1,258 | |
| #3 |  | **Search: Herbal medicine** (in Chinese) | | | 1,482 | |
| #4 |  | **Search: herbal** (in Chinese) | | | 333 | |
| #5 |  | **Search: herb** (in Chinese) | | | 89 | |
| #6 |  | **Search:** botanical (in Chinese) | | | 17,258 | |
| #7 | #1 OR #2 OR #3 OR #4 OR #5 OR #6 |  | | | 28,318 | |
| #8 |  | **Search: Acupuncture** (in Chinese) | | | 2,514 | |
| #9 |  | **Search:** acupuncture therapy (in Chinese) | | | 426 | |
| #10 |  | **Search:** needle acupuncture (in Chinese) | | | 392 | |
| #11 |  | **Search:** auricular acupuncture (in Chinese) | | | 73 | |
| #12 | #8 OR #9 OR #10 OR #11 |  | | | 2,774 | |
| #13 |  | **Search:** epilepsy (in Chinese) | | | 211 | |
| #14 |  | **Search:** pediatrics (in Chinese) | | | 3,033 | |
| #15 | #7 AND #12 AND #13 AND #14 |  | | | 17 | |
|  | Final | CHINESE | | | **(17)** | |
| **Chinese Biomedical Literature (CBM) (in Chinese)** | | | | | | |
| **R** | **Command** | **Strategies and keywords** | | | **Results** | |
| #1 |  | **Search: Traditional Chinese medicine** (in Chinese) | | | 72,915 | |
| #2 |  | **Search: Chinese herbal medicine** (in Chinese) | | | 5,009 | |
| #3 |  | **Search: Herbal medicine** (in Chinese) | | | 6,955 | |
| #4 |  | **Search: herbal** (in Chinese) | | | 11,143 | |
| #5 |  | **Search: herb** (in Chinese) | | | 4,177 | |
| #6 |  | **Search:** botanical (in Chinese) | | | 481 | |
| #7 | #1 OR #2 OR #3 OR #4 OR #5 OR #6 |  | | | 73,352 | |
| #8 |  | **Search: Acupuncture** (in Chinese) | | | 136,550 | |
| #9 |  | **Search:** acupuncture therapy (in Chinese) | | | 109,798 | |
| #10 |  | **Search:** needle acupuncture (in Chinese) | | | 134,911 | |
| #11 |  | **Search:** auricular acupuncture (in Chinese) | | | 822 | |
| #12 | #8 OR #9 OR #10 OR #11 |  | | | 115,454 | |
| #13 |  | **Search:** epilepsy (in Chinese) | | | 61,908 | |
| #14 |  | **Search:** pediatrics (in Chinese) | | | 33,310 | |
| #15 | #7 AND #12 AND #13 AND #14 |  | | | 0 | |
|  | Final | CHINESE | | | **(0)** | |
| **Wanfang Database (in Chinese)** | | | | | | |
| **R** | **Command** | **Strategies and keywords** | | | **Results** | |
| #1 |  | **Search: Traditional Chinese medicine** (in Chinese) | | | 1,773,043 | |
| #2 |  | **Search: Chinese herbal medicine** (in Chinese) | | | 786,200 | |
| #3 |  | **Search: Herbal medicine** (in Chinese) | | | 78,273 | |
| #4 |  | **Search: herbal** (in Chinese) | | | 54,080 | |
| #5 |  | **Search: herb** (in Chinese) | | | 1,186 | |
| #6 |  | **Search:** botanical (in Chinese) | | | 1,173,198 | |
| #7 | #1 OR #2 OR #3 OR #4 OR #5 OR #6 |  | | | 73,352 | |
| #8 |  | **Search: Acupuncture** (in Chinese) | | | 225,878 | |
| #9 |  | **Search:** acupuncture therapy (in Chinese) | | | 7,047 | |
| #10 |  | **Search:** needle acupuncture (in Chinese) | | | 139,125 | |
| #11 |  | **Search:** auricular acupuncture (in Chinese) | | | 2,684 | |
| #12 | #8 OR #9 OR #10 OR #11 |  | | | 213,767 | |
| #13 |  | **Search:** epilepsy (in Chinese) | | | 84,149 | |
| #14 |  | **Search:** pediatrics (in Chinese) | | | 393,383 | |
| #15 | #7 AND #12 AND #13 AND #14 |  | | | 103 | |
|  | Final | CHINESE | | | **(103)** | |
